# CRISPR/Cas9 Knockout of Bak Mediates Bax Translocation to Mitochondria in response to TNF*α*/CHX-induced Apoptosis

**DOI:** 10.1155/2019/9071297

**Published:** 2019-09-17

**Authors:** Jingtian Zhang, Han Niu, Zhizhuang Joe Zhao, Xueqi Fu, Yuxiang Wang, Xu Zhang, Fuqiang Zhang, Linlin Zeng

**Affiliations:** ^1^Edmond H. Fischer Signal Transduction Laboratory, College of Life Sciences, Jilin University, Changchun, 130012, China; ^2^Scientific Research Centre of China-Japan Union Hospital, Jilin University, Changchun, 130033, China; ^3^Department of Pathology, University of Oklahoma Health Sciences Center, Oklahoma City, OK, USA

## Abstract

TNF*α*/CHX-induced apoptosis is dependent on caspase-8 activation and regulated by Bcl-2. However, the specific participants and precise mechanisms underlying this apoptotic pathway are poorly understood. The proapoptotic proteins Bak and Bax—members of the Bcl-2 family—are essential for the functioning of the mitochondrial apoptotic pathway. In this study, we used the CRISPR/Cas9 system to knockout Bak in the human SH-SY5Y cell line and determined the effects of this knockout on TNF*α*/CHX-induced apoptosis. Our data showed that overexpression of Bcl-2 dramatically prevented TNF*α*/CHX-induced apoptosis, and then pro-apoptotic protein Bak was downregulated and became more resistant to TNF*α*/CHX-induced apoptosis, because both TNF*α*/CHX-induced PARP cleavage and caspase activation were blocked in BAK−/− cells or using specific siRNA, whereas Bax was dispensable in TNF*α*/CHX-induced apoptosis, as evidenced using specific siRNA. Bax translocated from the cytosol into the mitochondria in response to TNF*α*/CHX, and CRISPR/Cas9 knockout of Bak significantly decreased this translocation. These results indicate that TNF*α*/CHX-induced apoptosis does not occur in Bak−/− cells, suggesting that TNF*α*/CHX-induced apoptosis is Bak-dependent but Bax-independent.

## 1. Introduction

Tumor necrosis factor-*α* (TNF*α*)—a member of the family of death receptor ligands—induces cell death through the extrinsic death receptor apoptosis pathway. TNF*α* is the most essential inflammatory cytokine, primarily produced by macrophages, and it is involved in multiple cellular precesses, including proliferation, inflammation, and cytotoxic responses [1]. For instance, TNF*α* induced apoptosis in human gastric cancer cells [2]. Moreover, it promoted hepatocyte death [3]. In addition, TNF*α* is also critical for the survival of dendritic cells, and TOM-independent complex formation of Bax and Bak in mitochondrial fraction in TNF*α*-induced apoptosis in HeLa cells [4, 5]. FASL and TRAIL are other members of the family of death receptor ligands. FASL is the ligand of FAS (or CD95), which triggers cell death that depends upon caspase-8 activation. Although the molecular events spanning from receptor engagement until cell death have already been investigated in details, precise mechanisms underlying TNF*α*-induced apoptosis remain unclear.

Apoptosis, a form of programmed cell death, plays a crucial role in cellular differentiation, tissue homeostasis, and aging. Abnormal apoptosis regulation may be directly or indirectly associated with many diseases, such as cancer, autoimmune diseases, and neurodegenerative disorders. There are two major apoptotic pathways: the extrinsic death receptor apoptosis pathway and the intrinsic mitochondrial apoptosis pathway. The Bcl-2 family of proteins constitutes both pro- and antiapoptotic members, which maintain the mitochondrial integrity. The Bcl-2 family of proteins comprises three main groups classified according to Bcl-2 homology (BH) regions and function: (1) the multidomain antiapoptotic group (Bcl-2, Bcl-xl, and MCL-1); (2) the multidomain proapoptotic group (Bax and Bak); and (3) the BH3-only proapoptotic group (Bid, Bim, Bad, and Puma). Bak and Bax play pivotal roles in the regulation of mitochondrial permeability to trigger the release of mitochondrial proapoptotic factors, such as cytochrome C and Smac [6]. Additionally, mutations in the Bak gene have been indicated in human gastrointestinal cancers, and reduction in Bak protein levels has been observed in human gastric and colorectal tumors, indicating the critical role of Bak in pathogenesis of various tumors [7]. Furthermore, Bak-mediated apoptosis may contribute to TNF*α*-induced apoptosis; however, the molecular mechanisms underlying this apoptotic pathway remain unknown [8, 9].

The CRISPR/Cas9 system is a novel genetic technology derived from the prokaryotic adaptive immune system and directed by single-guide RNAs (sgRNA) for inducing specific DNA modifications in target genes [10]. sgRNA directs the target sequence to shear double-stranded DNA to modify specific sequences in genomic DNA. Thus, the CRISPR/Cas9 system is an important tool for targeted gene editing, which can repress gene expression [10] and knockin [11] or knockout genes [12]. This technology has widespread applications in drug development and personalized gene therapy for cancers, infectious diseases, and autoimmune disorders [13, 14].

In this study, we used siRNA and CRISPR/Cas9 to explore roles of Bak in TNF*α*/cycloheximide (CHX)-induced apoptosis and demonstrate that Bak was critical for this apoptosis. Our findings provide novel insights into mechanisms underlying TNF*α* cytotoxicity.

## 2. Materials and Methods

### 2.1. Reagents

Specific siRNAs and siRNAmate reagents were purchased from Gene Pharma (Shanghai, China). Complete Protease Inhibitor Cocktail Tablets were purchased from Bimake (Houston, TX). Western blot antibodies agonist PARP, caspase-3, and Bak were purchased from Cell Signaling Technology (Boston, MA). *β*-actin antibody was purchased from Santa Cruz Biotechnology (Stanta cruz, CA). Bax antibody was purchased from R&D Systems (Minneapolis, MN). Human TNF*α* were purchased from Novus Biologicals (Centennial, CO). Cytochrome C antibody was purchased from Selleckchem (Houston, TX). COX I antibody, goat anti-mouse IgG(H + L)/HRP, goat anti-Rabbit IgG(H + L)/HRP, and Rabbit Anti-Mouse IgG(H + L)/HRP were purchased from Bioss (Beijing, China). CHX was from Cayman Chemical (Beijing, China). The Mammalian Mitochondria Isolation Kit was purchased from TransGen Biotech (Beijing, China).

### 2.2. Cell Culture

Human neuroblastoma cells-SH-SY5Y, Human cervical cancer cells-HeLa, human colon cancer cells-SW 480, and human lung cancer cells-A549 were cultured in DMEM (Hyclone) supplemented with 10% fetal bovine serum (Gibco), 100 μg/ml streptomycin (Hyclone), and 100 units/ml penicillin (Hyclone). All of the cells were cultured in an incubator at 37°C and 5% CO_2_.

### 2.3. Western Blotting

Cells were harvested and lysed by sonication for 25 s on ice in lysis buffer [15]. For routine Western blotting, cell lysates were boiled at 100°C for 10 min, and the samples were subjected to SDS–PAGE (9% for PARP and *β*-actin and 10%–14% for Bax; Bak; Bid; cytochrome C; COX1; and caspase-3, -8, and -9). Samples were then transferred to polyvinylidene fluoride membranes probed with appropriate antibodies.

### 2.4. siRNA Treatment

Specific siRNAs for Bak and Bax were generated by Gene Pharma. A control-non specific siRNA duplex was also generated from Gene Pharma. Cells were transfected with Bak or Bax specific siRNAs twice at 2-day intervals using siRNAmate reagent, following the manufacturer's instructions. On day 5, cells were treated with TNF*α* and CHX. After 6 h of treatment, cells were harvested and lysed for further analysis.

### 2.5. Mitochondrial Preparation

For detection of the release of cytochrome C and Bak, and the translocation of Bax, cytosolic and mitochondrial extracts were prepared by permeabilization of cells using the Mitochondria Isolation Kit, following the manufacturer's instructions.

### 2.6. Flow Cytometry

After 6 h of treatment with TNF*α* and CHX, both attached and floating cells were harvested. According to the instruction of the Annexin V/PI Detection Kit, cells were stained with annexin V-enhanced green fluorescent protein/propidium iodide (PI) after washing twice with ice-cold PBS, and analyzed using the EpicsXL-MCL flow cytometer. Both annexin V- and PI-negative cells [annexin V−/PI−; quadrant 3] were considered normal survival cells; annexin V-positive and PI-negative and (annexin V+/PI−; quadrant 4) cells were thought to be in the early apoptotic stage; annexin V-and PI-positive (annexin V+/PI+; quadrant 2) cells were thought to be in the late apoptotic stage; annexin V-negative and PI-positive (annexin V−/PI+; quadrant 1) cells were considered mechanically injured cells.

### 2.7. CRISPR/Cas9

A CRISPR design website (http://crispr.mit.edu/) was used to design guide RNA for BAK knockout in SH-SY5Y cell. Then, 1# and 5# double oligonucleotides, with a length of 20-bp before the PAM site, were selected. These knocked out a 68-bp stretch in the Bak sequence. Finally, CACCG was added toward the 5′ end to generate a U6 promotor transcription recognition site, and CAAA was added toward the 3′ end to form sticky ends following BbsI digestion. Guide RNA sequences were as follows:

1#: 5′– CACCG GTTGATGTCGTCCCCGATGA–3′

3′–CCAACTACAGCAGGGGCTAC CAAA–5′

5#: 5′–CACCG TCATAGGCATTCTCTGCCGT–3′

3′–CAGTATCCGTAAGAGACGGCACAAA–5′

Bak guide RNA targeted exon 2 of the BAK gene. Oligonucleotides for guide RNAs were annealed with T4 ligase and cloned into the pX459-SpCas9-PX330-based plasmid (Addgene) using stbl 3 competent cells to amplify. Both 1# and 5# plasmids were transfected into SH-SY5Y cells using Lipofectamine^®^2000. After 2 days of transfection, growth medium was changed to selection medium containing 1 µg/ml puromycin. The knockouk effect of Bak was confirmed using western blot analysis.

## 3. Results and Discussion

### 3.1. Bcl-2 Overexpression Inhibits TNFα/CHX-induced Apoptosis

**Figure 1:**
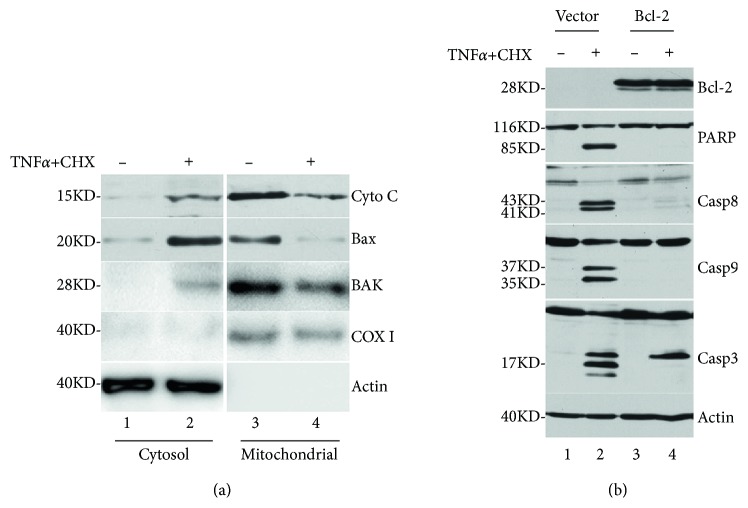
Overexpression of the antiapoptotic protein Bcl-2 inhibited TNF*α*/CHX-induced apoptosis. (a) Top panels show cytochrome c release from the mitochondria (right column) to the cytosol (left column). Second panels show Bax translocation from the cytosol (left panel) to the mitochondria (right panel). Third panels show Bak release from the mitochondria (right column) to the cytosol (left column). Fourth panels using antibody COX I to confirm the mitochondria remained intact using mitochondria isolation kit. Bottom panels show loading control *β*-actin. (b) After 24 h of transfection, Bcl-2 levels were significant (top panel, lanes 3 and 4). Western blots for PARP (second); caspase-8, -9, and -3 (panels 3–5); and loading control *β*-actin (bottom panel) are shown.

TNF*α* induces cell death through the extrinsic apoptosis pathway, however, Bcl2 family plays a critical role. First, translocation of Bax to the mitochondria, release of cytochrome c and Bak into the cytosol were also detected upon co-treatment of TNF*α*/CHX. After 5 h, we detected that cytochrome c and Bak release from the mitochondria to the cytosol, and Bax translocation from the cytosol to the mitochondria in SHSY5Y cells (see Figure 1(a)).

We further determined the effects of Bcl-2 family of proteins, which exhibit an antiapoptotic function, on TNF*α*/CHX-induced apoptosis to determine the mechanism underlying this apoptosis. Bcl-2 plasmids or an empty vector plasmid were transiently transfected into HeLa cells for 24 h, after which TNF*α*/CHX apoptosis was induced. In cells transfected with empty vectors, Bcl-2 expression was significant and PARP cleavage and caspase activation were observed (see Figure 1(b)). However, in the Bcl-2-transfected cells, TNF*α*/CHX-induced PARP cleavage and caspase activation were almost completely blocked (see Figure 1). These results clearly indicated that Bcl-2 overexpression inhibited TNF*α*/CHX-induced apoptosis in HeLa cells.

### 3.2. BAK, but not Bax, is Necessary for TNFα/CHX-induced Apoptosis

**Figure 2:**
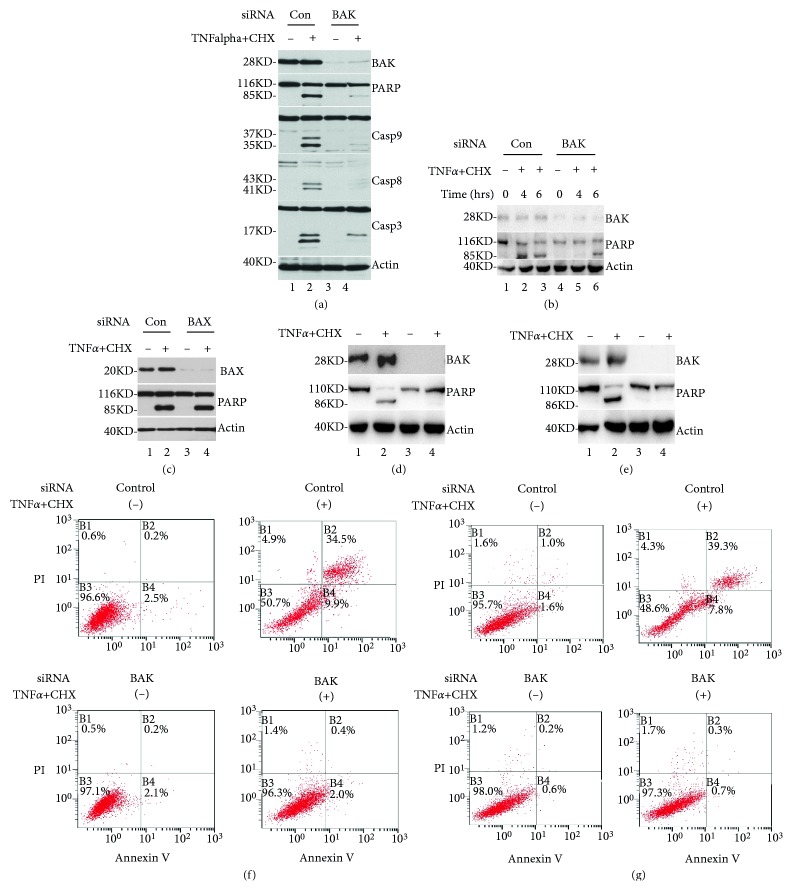
BAK knockdown, but Bax, blocked TNF*α*/CHX-induced apoptosis. (a) Bak knockdown inhibited TNF*α*/CHX-induced apoptosis in HeLa cells. After transfection of cells with non-specific siRNA (lanes 1 and 2) and specific Bak siRNA (lanes 3 and 4), cells were subjected to co-treatment with TNF*α*/CHX (+) for 5 h or left untreated (−). Western blots using antibodies against Bak (panel 1); PARP (panel 2); caspase-9, -8, and-3 (panels 3–5); and *β*-actin (bottom panel) are shown (relative sample loading). (b) Bak knockdown inhibited TNF*α*/CHX-induced apoptosis in SH-SY5Y cells. After transfection of SH-SY5Y cells with non-specific siRNA (lanes 1 and 2) and specific Bak siRNA (lanes 3 and 4), cells were subjected to co-treatment with TNF*α*/CHX (+) for 5 h and 7 h or left untreated (−). Western blots using antibodies against Bak (panel 1), PARP (panel 2), and *β*-actin (panel 3) are shown (relative sample loading). (c) Bax knockdown had no effect on TNF*α*/CHX-induced apoptosis in HeLa cells. After transfection with non-specific siRNA (lanes 1 and 2) and specific Bax siRNA (lanes 3 and 4) into HeLa cells, cells were subjected to co-treatment with TNF*α*/CHX (−) for 5 h or left untreated (−). Western blots using antibodies against Bak (panel 1), PARP (panel 2), and *β*-actin (panel 3) (relative sample loading). (d, e) Bak knockdown inhibited TNF*α*/CHX-induced PARP cleavage in A549 and SW480 cells. After transfection with non-silencing siRNA (lanes 1 and 2) and specific Bak siRNA (lanes 3 and 4) into A549 (d) and SW480(e) cells, cells were subjected to co-treatment with TNF*α*/CHX (−) for 6 h or left untreated (−). Western blots using antibodies against Bak (panel 1), PARP (panel 2), and *β*-actin (panel 3) (relative sample loading). (f, g) Non-silencing siRNA-transfected cells (top panels) and Bak-knockdown cells (bottom panels) were double stained with annexin V-enhanced green fluorescent protein and PI. Fluorescence was detected using a flow cytometer to analyze necrotic in the presence (right column) or absence (left column) of TNF*α* and CHX for 6 h in A549 (f) and SW480 (g) cells.

To further explore roles of Bax and Bak in TNF*α*/CHX-induced apoptosis, we detected the effects of Bax and Bak knockdown on TNF*α*/CHX-induced apoptosis. Hela cells were transfected with Bak-specific siRNAs; non-silenced siRNA-treated cells were used as controls. Bak was completely knocked down using siRNA (see Figure 2). Upon treatment with TNF*α*/CHX, PARP cleavage and caspases activation were almost blocked following Bak knockout (see Figure 2(a)). Interestingly, TNF*α*/CHX-induced apoptosis was not affected by Bax knockout (see Figure 2(b)). To further determine whether Bak-dependent TNF*α*/CHX-induced apoptosis was cell type-specific or generalizable to various cell types, effects of Bak knockdown on TNF*α*/CHX-induced apoptosis were assessed in SH-SY5Y cells. After co-treatment with TNF*α*/CHX for 5 and 7 h, Bak knockdown significantly inhibited TNF*α*/CHX-induced PARP cleavage at 5 h (see Figure 2(b)); however, this treatment only slightly affected TNF*α*/CHX-induced PARP cleavage at 7 h. Taken together, these results strongly indicated that TNF*α*/CHX-induced apoptosis was dependent on Bak but not on Bax.

To determine further whether Bak-dependent apoptosis induced by TNF*α* and CHX is a cell type-specific, we examined the effect of silencing Bax on TNF*α* and CHX-induced apoptosis in SW480 cells and A549 cells. Our data showed that knockdown of Bak had dramatic effect on TNF-alpha and CHX-induced PARP cleavage (see Figure 2(d) (A549) and 2(e) (SW480)). Furthermore, an Annexin/PI double staining assay was employed to examine cells in apoptotic/necrotic stages. 6 h after induction, cells were harvested and double stained with Annexin V/PI, in turn analyzing in a flow cytometry analyzer as described under “Materials and methods.” As shown in Figure 2(f) (A549) and 2(g) (SW480), the amounts of apoptosis/necrosis cells were about 45%, in TNF*α*/CHX-triggered (right column), A549 (top panel), and SW480 (third panel) cells as determined by the B4 (Annexin V+/PI−) and B2 (Annexin V+/PI+) cells. However, in the Bak specific siRNA-transfected cells (second and fourth panels), the basal levels of B4 (AnnexinV+/PI−) and B2 (AnnexinV+/PI+) cells were 2%. Taken together, these results clearly indicate that Bak play a crucial role on TNF*α*/CHX-induced apoptosis in the different types of cells.

### 3.3. Establishment of CRISPR/Cas9 Knockout of Bak in SH-SY5Y Cells

**Figure 3:**
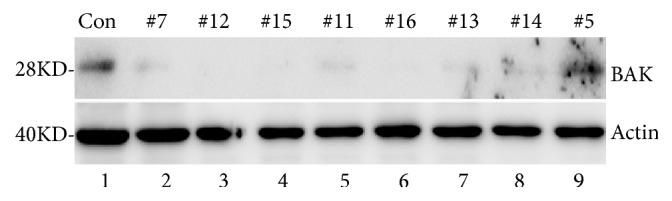
CRISPR/Cas9 knockout of Bak in SH-SY5Y cells. Western blots using Bak antibody for analysis of Bak knockout mutants are shown (top panel). Western blots using *β*-actin antibody are shown to indicate loading control (bottom panel).

To further confirm roles of Bak in TNF*α*/CHX-induced apoptosis, CRISPR/Cas9 was used to establish a stable Bak-knockout SH-SY5Y cell line. SH-SY5Y cells were transfected with a plasmid expressing guide RNA against the Bak sequence to generate Bak-knockout cells. The plasmid expressing non-targeting guide RNA was used to generate parallel control cells with Bak. Efficiency of Bak knockout in SH-SY5Y cells is shown in Figure 3. Compared to that in with controls, Bak expression in a few samples was significantly reduced in the cells transfected with plasmid expressing the Bak guide RNA (see Figure 3), indicating a successful Bak knockout in most SH-SY5Y cells. Based on this result, we selected clones #12, #15, and #16 for subsequent experiments and #5 as a control.

### 3.4. Bak is Required for TNFα/CHX-induced PARP Cleavage, caspase-3 Activation, Cytochrome C Release, and Bax Translocation in Bak-KO Cells

**Figure 4:**
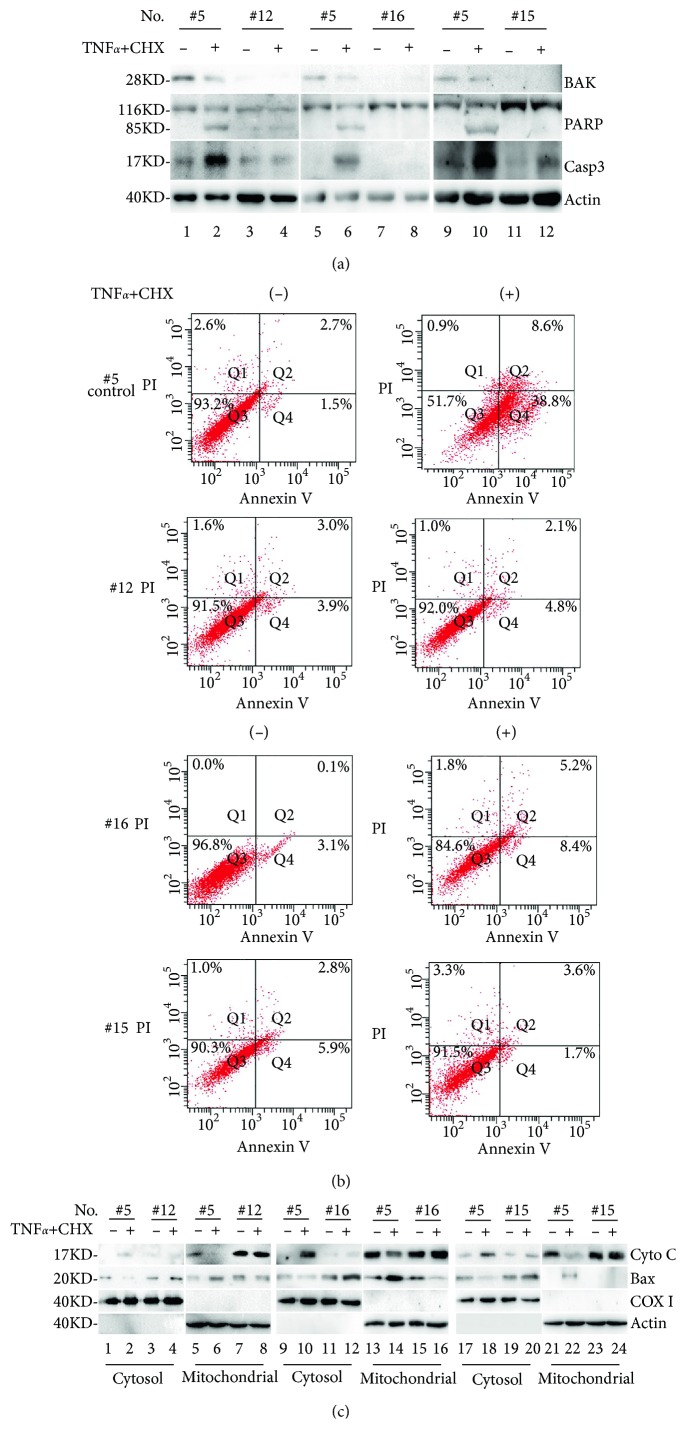
Bak knockout inhibited TNF*α*/CHX-induced PARP cleavage generation, caspase-3 activation, cytochrome C release, and Bax translocation. After induction of TNF*α* and CHX for 6 h, CRISPR control cells (#5) and Bak-knockout cells (#12, #16, and #15) were harvested and divided into two batches. One batch of cells were directly lysed and subjected to SDS–PAGE, in turn followed by Western blotting. The other batch was used for preparation of cytosolic and mitochondrial fractions using the Mitochondria Isolation Kit. (A) Bak expression (panel 1), PARP cleavage (panel 2), and caspase-3 activation (panel 3) in various cells is shown. Membranes in top panels were reprobed with *β*-actin antibody to indicate relative samples loading (bottom panels). (B) CRISPR control cells (#5, row 1) and Bak-KO cells (#12, #16, and #15, rows 2–4) were double stained with annexin V and PI. Fluorescence was detected using a flow cytometer to analyze necrotic (annexin−/PI+, quadrant 1), nonapoptotic (annexin−/PI−, quadrant 3), early apoptotic (annexin+/PI−, quadrant 4), and late apoptotic cells (annexin+/PI+, quadrant 2) in the presence (right column) or absence (left column) of TNF*α* and CHX for 6 h. (C) Cytochrome C (panel 1) from the mitochondria into the cytosol and Bax translocation (panel 2) from the cytosol into the mitochondria are shown. Membranes in top panels were reprobed with COX I antibody for confirming the mitochondrial integrity during experimental (panel 3). Membranes of second panel were reprobed with *β*-actin antibody to indicate relative sample loading of cytosolic fractions (bottom panels).

We next examined whether Bak knockout affected TNF*α*/CHX-induced apoptosis in Bak-knockout cells. BAK was almost completely knocked out in #12, #16, and #15 cells using the CRISPR/Cas9 system (see Figure 4(a)). Compared with those in controls, TNF*α*/CHX-induced PARP cleavage and caspase-3 activation were blocked in #12, #16, and #15 Bak-knockout cells (see Figure 4(a)).

To further confirm effects of Bak on TNF*α*/CHX-induced apoptosis, annexin V/PI staining assay was used to detect cells in early and late apoptotic phases and necrotic stages. Upon induction of TNF*α*/CHX, proportions of early and late apoptotic/necrotic cells were nearly 38.8% and 9.5%, respectively (see Figure 4(b)). In #12, #16, and #15 Bak-knockout cells, the basal levels of Q4 (annexin V+/PI−) and Q2 (annexin V+/PI+) cells were <9% and <6%, respectively, upon co-treatment with TNF*α*/CHX (see Figure 4(b)). In untreated cells, the basal levels of Q4 (annexin V+/PI−) and Q2 (annexin V+/PI+) cells were <6% and <3%, respectively, in control (#5) and Bak-knockout cells. Taken together, these results strongly suggest that Bak is required for TNF*α*/CHX-induced apoptosis in SH-SY5Y cells.

TNF*α*/CHX can activate the mitochondrial signaling pathway, leading to cytochrome C release into the cytosol and Bax translocation into the mitochondria [6]. To investigate roles of Bak in TNF*α*/CHX-induced apoptosis, we assessed effects of Bak on TNF*α*/CHX-induced cytochrome C release and BAX translocation. In control (#5) cells, cytochrome C was released from mitochondria into cytosol and BAX translocated from cytosol into mitochondria upon induction of TNF*α* and CHX (see Figure 4(c)). However, cytochrome C release and BAX translocation were completely blocked by BAK knockout in #12, #15, and #16 cells (see Figure 4(c)).

Collectively, these results clearly indicated that TNF*α*/CHX-induced PARP cleavage generation, caspase-3 activation, cytochrome C release, and Bax translocation were Bak dependent.

## 4. Discussion

In this study, we used the CRISPR/Cas9 system to explore roles of Bak in TNF*α*/CHX-induced apoptosis in SH-SY5Y cells, and Bak is essential for TNF*α*/CHX-induced PARP cleavage, caspase-3 activation, cytochrome C release, and Bax translocation via annexin V/PI staining and Western blotting, suggesting that Bak played a critical role in TNF*α*/CHX-induced apoptosis and propose a new evidence to study the mechanism and target for protection against TNF*α*/CHX-induced cell death.

TNF*α* is a pleiotropic cytokine regulating multiple cellular processes, such as inflammation, cell survival, and apoptosis [1, 2], and it serves diverse functions in multiple cell types. Reportedly, TNF*α*/CHX induced apoptosis in gastric cancer cells MKN28, which was triggered by accelerated degradation of IAP family proteins in addition to inhibition of the NF-kB-dependent synthesis of antiapoptotic molecules [2].

The lysosomal degradation product of ceramide, sphingosine, was required for TNF*α*/CHX-induced apoptosis in hepatoma cells via induction of lysosomal membrane permeabilization [16]. A prostate-specific homeobox gene, NKX3.1, could potentiate TNF*α*/CHX-induced apoptosis in prostate cancer PC-3 cells and LNCaP cells via caspase-3 activation [17].

Furthermore, the proapoptotic protein Bak, which is involved in apoptotic execution, undergoes conformational changes during this process. Although the mechanisms underlying apoptotic regulation by different members of the Bcl-2 family have been shown to be both complex and diverse, roles of different mitochondrial receptors of the Bcl-2 family in apoptosis have not been completely elucidated to date [18, 19]. Bak interacts with Metaxin 1 upon Mtx1 phosphorylation during death receptor-induced apoptosis [6]. Similarly, Bak levels significantly increased after co-stimulation of CHX/lexatumumab-triggered apoptosis [20, 21]. In addition, the chemotherapeutic drug paclitaxel-induced apoptosis was Bak-dependent, indicating that Bak may be a prognostic marker or therapeutic target to determine and overcome paclitaxel sensitivity and resistance in human breast cancer [22]. *Coptidis rhizoma* water extract elicited anticancer effects via Bax and Bak activation by triggering apoptosis in human melanoma cells [23]. TNF*α*-induced necroptosis required the RIP-1/RIP-3 necrosome, while death was mediated by Bax/Bak [8].

Furthermore, Bax/Bak played a critical role in inflammation-induced necroptosis triggered by various stimuli, such as induction of TNF*α*/cycloheximide/Z-VAD-fmk or Smac mimetic BV6/dexamethasone [24].

During disease-induced chronic inflammation, such as that in periodontal disease, TNF*α* and CHX led to altered bone remodeling. We investigated effects of TNF*α*/CHX on apoptosis in SH-SY5Y and HeLa cells and observed that co-treatment with TNF*α* and CHX reduced cell viability and triggered cell apoptosis, as indicated by PARP cleavage and caspase-3 activation. Western blotting revealed that TNF*α*/CHX induced cytochrome c release and Bax translocation.

Bcl-2 overexpression significantly attenuated cellular apoptosis following co-treatment with TNF*α*/CHX. Upon examination of the role of Bax and Bak in TNF*α*/CHX-induced apoptosis, we demonstrated that TNF*α*/CHX-induced apoptosis was dependent on Bak, but not on Bax. Furthermore, Bak knockout inhibited TNF*α*/CHX-induced PARP cleavage, caspase-3 activation, cytochrome C release, and Bax translocation.

## 5. Conclusions

In conclusion, Bak knockout inhibited TNF*α*/CHX-induced apoptosis. Our study further underlines the potential applications of CRISPR/Cas9 system in drug screening and targeted therapeutics.

## Data Availability

The data used to support the findings of this study are available from the corresponding author upon request.

## References

[1] Faustman D., Davis M. (2010). TNF receptor 2 pathway: drug target for autoimmune diseases. *Nature Reviews Drug Discovery*.

[2] Kitagawa M., Shiozaki A., Ichikawa D. (2015). Tumor necrosis factor-alpha-induced apoptosis of gastric cancer MKN28 cells: accelerated degradation of the inhibitor of apoptosis family members. *Archives of Biochemistry and Biophysics*.

[3] Minero V. G., Khadjavi A., Costelli P., Baccino F. M., Bonelli G. (2013). JNK activation is required for TNFalpha-induced apoptosis in human hepatocarcinoma cells. *International Immunopharmacology*.

[4] Ross K., Rudel T., Kozjak-Pavlovic V. (2009). TOM-independent complex formation of Bax and Bak in mammalian mitochondria during TNFα-induced apoptosis. *Cell Death and Differentiation*.

[5] Lehner M., Kellert B., Proff J. (2012). Autocrine TNF is critical for the survival of human dendritic cells by regulating BAK, BCL-2, and FLIPL. *The Journal of Immunology*.

[6] Petit E., Cartron P. F., Oliver L., Vallette F. M. (2017). The phosphorylation of Metaxin 1 controls Bak activation during TNFalpha induced cell death. *Cellular Signalling*.

[7] Wang G.-Q., Gastman B. R., Wieckowski E. (2001). A role for mitochondrial Bak in apoptotic response to anticancer drugs. *Journal of Biological Chemistry*.

[8] Tischner D., Manzl C., Soratroi C., Villunger A., Krumschnabel G. (2012). Necrosis-like death can engage multiple pro-apoptotic Bcl-2 protein family members. *Apoptosis*.

[9] Kepp O., Rajalingam K., Kimmig S., Rudel T. (2007). Bak and Bax are non-redundant during infection- and DNA damage-induced apoptosis. *The EMBO Journal*.

[10] Zhang R., Miner J. J., Gorman M. J. (2016). A CRISPR screen defines a signal peptide processing pathway required by flaviviruses. *Nature*.

[11] Zhou J., Wang C., Zhang K. (2016). Generation of human embryonic stem cell line expressing zsGreen in cholinergic neurons using CRISPR/Cas9 system. *Neurochemical Research*.

[12] Shi L., Meng T., Zhao Z. (2017). CRISPR knock out CTLA-4 enhances the anti-tumor activity of cytotoxic T lymphocytes. *Gene*.

[13] Yin H., Song C. Q., Dorkin J. R. (2016). Therapeutic genome editing by combined viral and non-viral delivery of CRISPR system components in vivo. *Nature Biotechnology*.

[14] Zuckermann M., Kawauchi D., Gronych J. (2017). Applications of the CRISPR/Cas9 system in murine cancer modeling. *Briefings in Functional Genomics*.

[15] Zeng L., Li T., Xu D. C. (2012). Death receptor 6 induces apoptosis not through type I or type II pathways, but via a unique mitochondria-dependent pathway by interacting with Bax protein. *The Journal of Biological Chemistry*.

[16] Ullio C., Casas J., Brunk U. T. (2012). Sphingosine mediates TNFalpha-induced lysosomal membrane permeabilization and ensuing programmed cell death in hepatoma cells. *Journal of Lipid Research*.

[17] Pengju Z., Weiwen C., Aiying W. (2010). NKX3.1 potentiates TNF-alpha/CHX-induced apoptosis of prostate cancer cells through increasing caspase-3 expression and its activity. *Biochemical and Biophysical Research Communications*.

[18] Huai J., Vogtle F. N., Jockel L. (2013). TNFalpha-induced lysosomal membrane permeability is downstream of MOMP and triggered by caspase-mediated NDUFS1 cleavage and ROS formation. *Journal of Cell Science*.

[19] Pucci B., Bertani F., Karpinich N. O. (2008). Detailing the role of Bax translocation, cytochrome c release, and perinuclear clustering of the mitochondria in the killing of HeLa cells by TNF. *Journal of Cellular Physiology*.

[20] Zhao X., Cao M., Liu J. J., Zhu H., Nelson D. R., Liu C. (2011). Reactive oxygen species is essential for cycloheximide to sensitize lexatumumab-induced apoptosis in hepatocellular carcinoma cells. *PLoS One*.

[21] Luo P., Zhao Y., Li D. (2012). Protective effect of Homer 1a on tumor necrosis factor-alpha with cycloheximide-induced apoptosis is mediated by mitogen-activated protein kinase pathways. *Apoptosis*.

[22] Miller A. V., Hicks M. A., Nakajima W., Richardson A. C., Windle J. J., Harada H. (2013). Paclitaxel-induced apoptosis is BAK-dependent, but BAX and BIM-independent in breast tumor. *PLoS One*.

[23] Xu X., Yokoyama S., Hayakawa Y., Saiki I. (2017). Coptidis Rhizoma induces intrinsic apoptosis through BAX and BAK activation in human melanoma. *Oncology Reports*.

[24] Rohde K., Kleinesudeik L., Roesler S. (2017). A Bak-dependent mitochondrial amplification step contributes to Smac mimetic/glucocorticoid-induced necroptosis. *Cell Death and Differentiation*.

